# Isoflavone-Enriched Soybean Leaves (Glycine Max) Alleviate Cognitive Impairment Induced by Ovariectomy and Modulate PI3K/Akt Signaling in the Hippocampus of C57BL6 Mice

**DOI:** 10.3390/nu14224753

**Published:** 2022-11-10

**Authors:** Dae Young Yoo, Soonwoong Jung, Jae Soon Kang, Ji Hyeong Baek, Ki Hun Park, Dong Hoon Lee, Sang Soo Kang, Hyun Joon Kim

**Affiliations:** 1 Department of Anatomy and Convergence Medical Science, College of Medicine, Gyeongsang National University, Jinju 52727, Korea; 2 Institute of Health Science, Gyeongsang National University, Jinju 52727, Korea; 3 Tyrosine Peptide Multiuse Research Group, Gyeongsang National University, Jinju 52727, Korea; 4 Anti-Aging Bio Cell Factory Regional Leading Research Center, Gyeongsang National University, Jinju 52828, Korea; 5 Division of Applied Life Science (BK21 Plus), IALS, Gyeongsang National University, Jinju 52828, Korea

**Keywords:** menopause, phytoestrogen, isoflavone, cognition, ovariectomy

## Abstract

(1) Background: The estrogen decline during perimenopause can induce various disorders, including cognitive impairment. Phytoestrogens, such as isoflavones, lignans, and coumestans, have been tried as a popular alternative to avoid the side effects of conventional hormone replacement therapy, but their exact mechanisms and risk are not fully elucidated. In this study, we investigated the effects of isoflavone-enriched soybean leaves (IESLs) on the cognitive impairment induced by ovariectomy in female mice. (2) Methods: Ovariectomy was performed at 9 weeks of age to mimic menopausal women, and the behavior tests for cognition were conducted 15 weeks after the first administration. IESLs were administered for 18 weeks. (3) Results: The present study showed the effects of IESLs on the cognitive function in the OVX (ovariectomized) mice. Ovariectomy markedly increased the body weight and fat accumulation in the liver and perirenal fat, but IESL treatment significantly inhibited them. In the behavioral tests, ovariectomy impaired cognitive functions, but administration of IESLs restored it. In addition, in the OVX mice, administration of IESLs restored decreased estrogen receptor (ER) β and PI3K/Akt expression in the hippocampus. (4) Conclusions: The positive effects of IESLs on cognitive functions may be closely related to the ER-mediated PI3/Akt signaling pathway in the hippocampus.

## 1. Introduction

Menopause is a common physiological phenomenon characterized by the exhaustion of the primordial ovarian follicles and decreased estrogen levels [1]. The decline in estrogen during perimenopause can induce various disorders such as Alzheimer’s disease, cognitive impairment, and osteoporosis, as well as metabolic disorders, including insulin resistance and cardiovascular disease [2,3,4]. Menopause occurs on average at age 51 [5], so women live approximately 30 years after menopause. Therefore, to improve women’s quality of life, it is essential to establish prevention and therapeutic strategies for menopausal-related diseases.

Estrogen is not only associated with reproductive function but is also essential in regulating neuronal functions, including their proliferation, survival, and synaptic plasticity [6]. In addition, estrogen is involved in neurotransmission, which modulates mood and memory function in the central nervous system [7]. Several types of estrogen receptors (ERs) are expressed in various areas of our body, including the brain, and the interactions of estrogen and its receptors not only regulate gene transcription but also modulate multiple cellular signals, which are closely involved in synaptic plasticity [8]. In the hippocampal CA1 region of female rats, it was reported that estrogen mediated synaptogenesis and enhanced memory function [9]. In the human brain, estrogen deficiency causes structural abnormalities and functional disorders, eventually leading to degenerative disease [10]. In addition, it has been reported that memory is directly associated with the expression level of the ERs that bind to estrogen [11]. ERs bound by specific agonists activate kinase signals, and activation of these signals acts in a dose-dependent manner, playing an important role in mitigating cognitive decline or enhancing memory formation [11,12].

Estrogen and ERs-mediated actions are involved in the onset of various diseases, so hormonal replacement therapy (HRT) is widely used to minimize the symptoms that appear in menopause in women. Previous studies have reported that HRT reduced the occurrence and mortality of cardiovascular disease [13,14] and enhanced cognitive function in postmenopausal women [15]. Phytoestrogens, nonsteroidal plant compounds, are structurally similar to the steroid estrogen of vertebrates and have been used recently as a popular alternative to avoid the side effects of conventional HRT [16,17]. Isoflavones are plant chemicals belonging to phytoestrogens and have also been used as alternative therapies for various hormone-dependent diseases, including menopausal symptoms, cardiovascular disease, osteoporosis, and cancers [18]. In our previous study, we observed the anti-obesity effects of isoflavone-enriched soybean leaves (IESLs), produced by adding ethephon to soybean trees at the R3 stage, on ovariectomized (OVX) Sprague–Dawley rats, and confirmed that the IESLs were comprised of several types of isoflavones such as daidzin, malonyldiadzin, daidzein, genistin, malonylgenistin, and genistein [19,20].

Researchers have reported many beneficial aspects of phytoestrogens in various disorders, including menopause, aging, and cancer, via their anti-inflammatory and antioxidative effects [21,22]. Still, their exact mechanisms and risk are not fully elucidated; hence, the effects and safety of phytoestrogens should be re-evaluated. In the present study, IESL, which exhibited an anti-obesity effect in the previous study [23], was administered to ovariectomized mice to evaluate its effects on the cognitive decline induced in postmenopausal women.

## 2. Materials and Methods

### 2.1. Preparation of Isoflavone-Enriched Soybean Leaves (IESLs)

IESLs, produced by the method of Yuk et al. [24], were provided by Ki Hun Park’s lab (Gyeongsang National University, Jinju, Korea) [23]. These isoflavone-enriched soybeans were grown in a glasshouse for 2 months to reach a maximum growth stage of R3. At this stage, pod development was detected, and 250 μg/mL of ethephon was sprayed until dripping twice every 24 h. Four days later, the IESL were harvested, chopped, and dried at 35 °C. The dried leaves were mixed with water at a ratio of 1:10 and extracted at 100 °C for 6 h. Extracts of IESLs were adjusted to a final isoflavone concentration of 15 mg/g, and these extracts were used in this study.

### 2.2. Animals and Treatment

**Figure 1:**
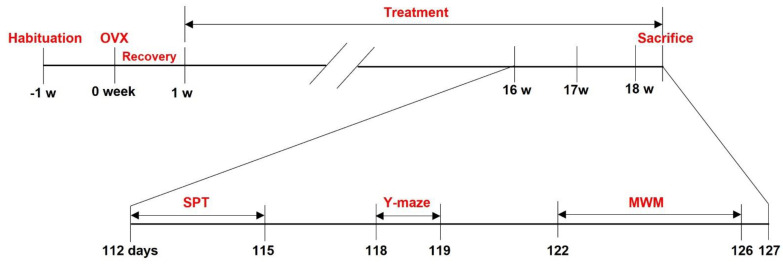
Schematic drawing of the experimental design.

Eight-week-old female C57BL/6 mice were purchased from Koatech, Co. Ltd. (Pyeongtaek, Korea). After one week of adaptation, a bilateral ovariectomy was performed as described previously [25]. In the study, we set the average weight of the mouse to 28 g. The IESL extract contained isoflavone at a concentration of 15 mg/g, and IESL extracts were mixed with compound feed to make chew blocks (Uni Faith, Seoul, Korea). The chew blocks were prepared to contain 11.7 g of IESL extract per 1 kg. As a result, the mice ate 18.8 mg/kg of isoflavone per day, which was the same concentration that confirmed the anti-obesity effect in the previous study [23]. The experimental scheme is provided in Figure 1. The mice were divided into three groups as the non-ovariectomized mice (Control, CTL; n = 10), the OVX mice (n = 9), and the OVX mice with IESL extracts (OVX + IESL; n = 10). Ovariectomy was performed at 9 weeks of age, and the behavioral tests were conducted 15 weeks after the first administration. IESLs were administered for about 18 weeks, after which the mice were sacrificed. All mice were housed in a room with constant temperature and humidity control (lights on 06:00–18:00) during the experimental period, with food and water available ad libitum. Body weight and food intake were checked every other day. All experimental procedures followed the National Institutes of Health guidelines and were approved by the Gyeongsang National University Institutional Animal Care & Use Committee (Approval no. GLA-100917-M0093).

Ovariectomy was performed at 9 weeks of age, and the behavioral tests were conducted 15 weeks after the first administration. IESL was administered for about 18 weeks, after which the mice were sacrificed.

### 2.3. Staining

#### Hematoxylin and Eosin (H&E) Staining

The mice were transcardially perfused with 4% paraformaldehyde, and dehydration and clearing of liver tissues were performed in a series of ethanol and xylene. Then, the liver tissues were embedded with paraffin wax using a Leica tissue processor (Leica TP 1020, Wetzlar, Germany), and they were sectioned by 5 µm with a microtome (Leica RM2235, Wetzlar, Germany). H&E staining was performed with standard protocols [26]. All images were obtained under a BX51 light microscope (Olympus, Hamburg, Germany).

### 2.4. Oil Red O Staining

Oil Red O staining was performed as described previously [23,27]. The fixed liver tissue was cryopreserved in 30% sucrose until the tissue sank and embedded in the embedding medium (OCT compound, Sakura Fineteck USA, Inc., Torrance, CA, USA) before cryosectioning. The tissues were sectioned by 10 µm with a cryostat (Leica CM 1950, Wetzlar, Germany) and Oil Red O stained with an Oil Red O kit (Abcam, ab150678, Cambridge, UK) for 40 min. All stained images were obtained with a BX51 light microscope.

### 2.5. Behavioral Assessments

#### Morris Water Maze Test

The Morris water maze test (MWM) was performed as previously described [28]. On the training days, the mice were positioned in a 120-cm circled swimming pool and trained to find an invisible platform in the pool. The water in the maze was maintained at 24 ± 1 °C and the light was 200 lx. For four consecutive days of acquisition, four trials were performed a day for each mouse. On day 5, the platform was removed, and the time spent in the quadrant where the flatform had been placed during the training days was calculated. The movement of each mouse was traced and analyzed with EthoVision (Noldus Information Technology, Wageningen, The Netherlands).

### 2.6. Sucrose Preference Test

As previously described, the sucrose preference test (SPT) was conducted to observe stress-induced anhedonia symptoms [29]. The mice were trained to taste 0.1 M sucrose solution for the first 48 h but were prohibited from ingesting the sucrose solution for the next 24 h. Then, the sucrose solution and water were placed in identical bottles, and the amount of drinking was observed for 6 h. The sucrose preference was provided as the ratio of sucrose-to-water consumption.

#### Y-Maze Test

The Y-maze test was performed to evaluate the exploratory behavior and working memory. To reduce the transport-related stress, animals in cages were brought to the animal behavior test room 30 min prior to all tests. Each mouse was placed in one arm (termed as B, always) facing opposite to the center of the maze and allowed to move through the apparatus for 8 min. Entry into an arm was considered only when all four paws were inside the arm. The first entry into B was excluded from the calculation. Spontaneous alternation (%) was defined as consecutive entries in the three different arms, divided by the total number of possible triads (total arm entries-2) [30]. The movement was analyzed with EthoVision.

### 2.7. Western Blot Analysis

Western blot analysis was conducted as previously described [31]. Proteins (10 µg each) were separated by SDS-PAGE and transferred to nitrocellulose membranes. Protein blocking was performed with 1% bovine serum albumin and 5% skim milk. Membranes were incubated with PI3K (1: 1000, ab40755, Abcam, Cambridge, UK), Akt (1:1000, 9272S, Cell Signaling, Danvers, MA, USA), pAkt (1:1000, 9271S, Cell Signaling, Danvers, MA, USA), ER-alpha (1:1000, SC-8002, Santa Cruz, Dallas, TX, USA), ER-beta (1:1000, SC-373853, Santa-Cruz, Dallas, TX, USA), and GAPDH (1:10,000, ab128915, Abcam, Cambridge, UK) primary antibodies. Antibody interactions were visualized with an enhanced chemiluminescence detection kit (Amersham Biosciences, Munich, Germany). The density for each band was quantified by Sigma Gel software (Sigma-Aldrich, St. Louis, MO, USA) to analyze the results. Each density was normalized using the corresponding GAPDH as an internal control.

### 2.8. Statistical Analysis

In the present study, the data were represented as the mean ± S.E.M. To investigate the effects of the IESLs on OVX mice, the differences in the mean value were analyzed statistically by one-way analysis of variance (ANOVA), followed by Bonferroni’s post hoc test with GraphPad Prism 5 (GraphPad Software, Inc., La Jolla, CA, USA). Statistical significance was considered when the *p* value was < 0.05.

## 3. Results

### 3.1. Establishment of the Mouse OVX Model and the Effects of IESLs on the Body Weight and Fat Accumulation

**Figure 2:**
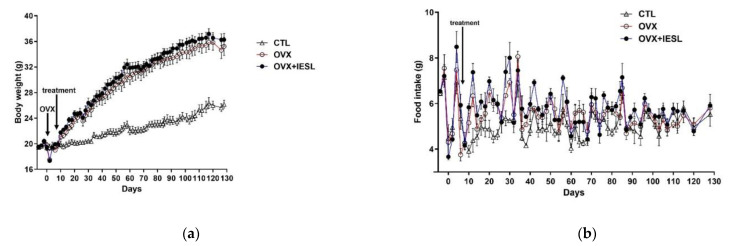

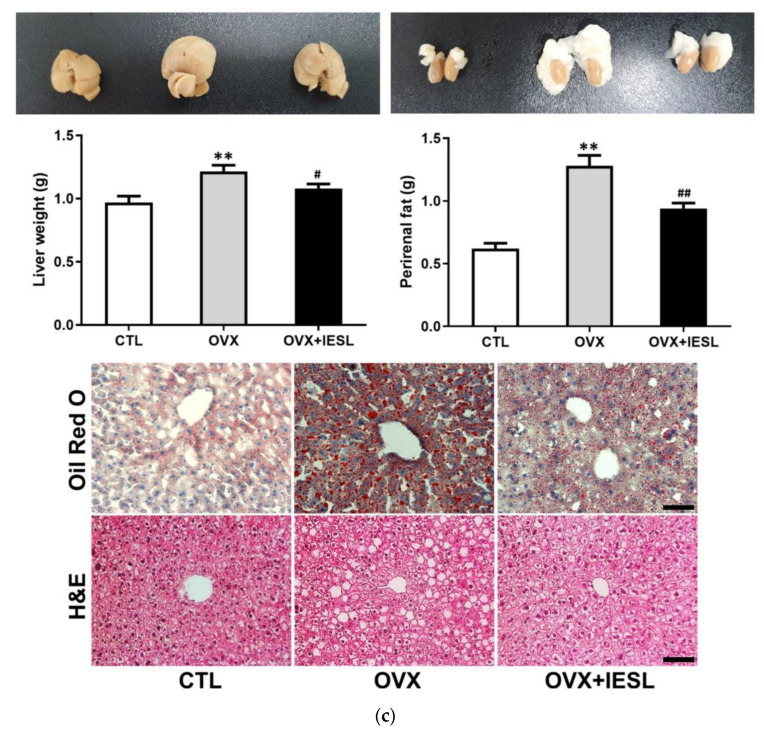
Establishment of the mouse ovariectomized (OVX) model and effects of the isoflavone-enriched soybean leave (IESL) extracts on the body weight, food intake, and fat accumulation in the control (CTL), OVX, OVX+IESL mouse groups. OVX increased the body weight significantly in the OVX and OVX+IESL groups compared to the CTL group (**a**), but there was no significant difference in food intake among the groups (**b**). In addition, OVX markedly increased the fat accumulation in the liver and perirenal tissues in the OVX+IESL group (**c**). However, administration of IESLs significantly decreased the fat accumulation compared with the OVX group. ** *p* < 0.01 vs. the CTL group; # *p* < 0.05 and ## *p* < 0.01 vs. the OVX group. Scale bars = 100 um.

The body weights steadily increased with aging in all groups (Figure 2a). At the end of the study, however, there was a significant increase in the OVX and OVX+IESL groups compared to the CTL group. The mean body weights of the OVX and OVX+IESL groups were 35.23 and 36.26, respectively. Unlike the body weight, however, there was no significant difference in food intake among the groups (Figure 2b). As described in the previous study [23], in the OVX group, the liver weight was significantly increased compared to the CTL group, and clearly increased fat deposition was observed in the hepatocytes in H&E and Oil Red O staining (Figure 2c). In addition, the perirenal fat mass was also significantly increased in the OVX group, but the administration of IESLs markedly inhibited the accumulation of lipid droplets in the liver and perirenal region.

### 3.2. Effects of the IESL Extracts on Behavioral Changes in the Morris Water Maze, Y-Maze, and Sucrose Preference Tests

**Figure 3:**
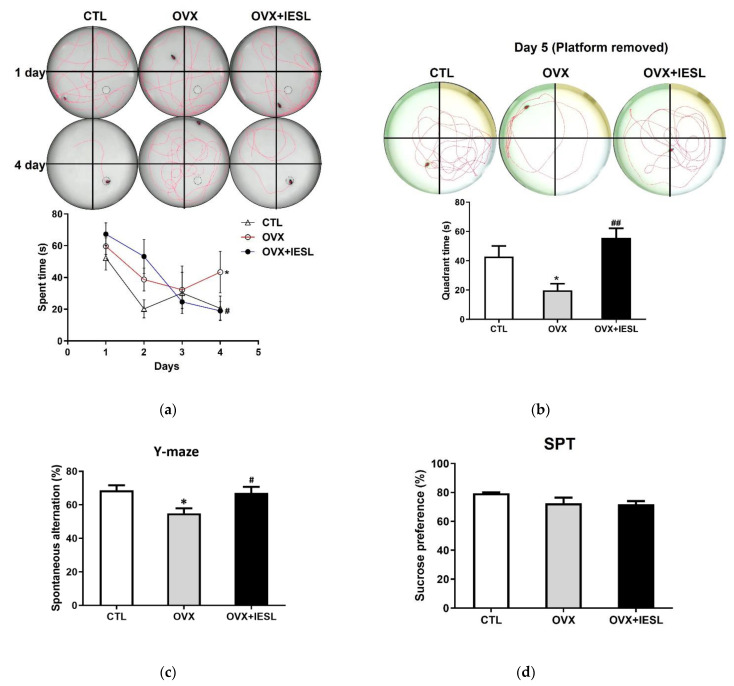
Effects of the IESL extracts on behavioral changes in the Morris water maze (**a**,**b**), Y-maze (**c**), and sucrose preference (**d**) tests. Representative individual swim paths and escape latency during four acquisition days (**a**), and representative individual swim paths and comparison of time spent in the target quadrant after removing the platform on day 5 (**b**). Spontaneous alternation (%) in the Y-maze test (**c**) and the sucrose preference test (SPT) in the CTL, OVX, and OVX+IESL groups (**d**). Data are expressed as the mean ± S.E.M. * *p* < 0.05 vs. the CTL group; # *p* < 0.05 and ## *p* < 0.01 vs. the OVX group.

The MWM test was performed to determine the effects of IESLs on the long-term and spatial memory deficit induced by ovariectomy. In the OVX group, the escape latencies and swimming distance were significantly increased during the training trials (Figure 3a), and the time spent in the target quadrant was decreased compared to those in the CTL group on the test day (Figure 3b). In contrast, the administration of IESLs significantly decreased the escape latency and swimming distance and increased the spent time in the target quadrant.

The Y-maze test was conducted to investigate the effects of IESL administration on the short-term and working memory. In the CTL group, the mean of spontaneous alternation was 68.63%, and the OVX group significantly decreased to 54.94% (Figure 3c). However, in the OVX+IESL group, IESL administration restored it to 67.07%.

### 3.3. Effects of the IESL Extracts on the Expression of ERs and the PI3K/Akt Pathway

**Figure 4:**
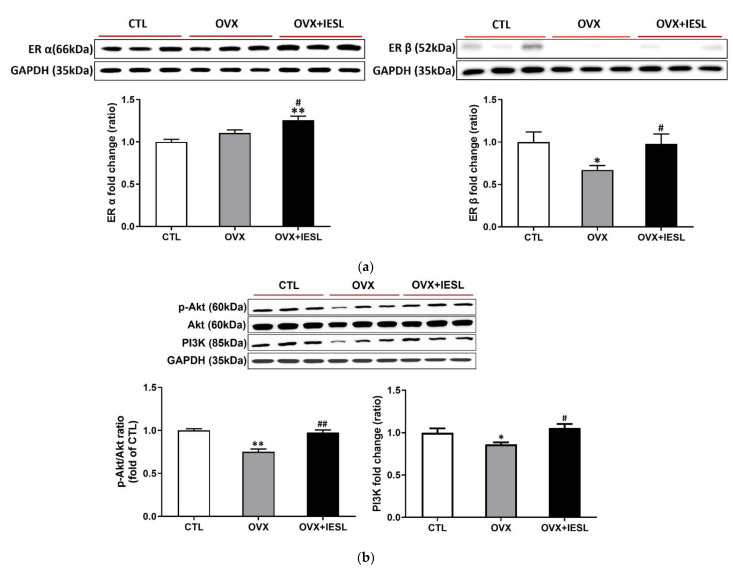
Effects of the IESL extracts on the expression of ERs and the PI3K/Akt pathway. Ovariectomy did not induce a significant change in the expression of ER α but decreased the expression of ER β (**a**). In the OVX+IESL group, the administration of IESL significantly increased both ERs compared to the OVX group. In the OVX group, expression of the PI3K and p-Akt/Akt ratio are significantly decreased compared with the CTL group, but the treatment of IESL restored them in the OVX+IESL group (**b**). Data are expressed as the mean ± S.E.M. * *p* < 0.05 and ** *p* < 0.01 vs. the CTL group; # *p* < 0.05 and ## *p* < 0.01 vs. the OVX group.

Western blotting for ERs and PI3/Akt was performed with hippocampal homogenates to evaluate the effects of ovariectomy and IESLs on the expression of ERs and their related cellular pathways. The ovariectomy did not induce a significant change in the expression of ER α but decreased the expression of ER β. In the OVX+IESL group, the administration of IESL significantly increased both ERs compared to the OVX group (Figure 4a). In the OVX group, the expression of the PI3K and p-Akt/Akt ratio decreased considerably compared to the CTL group, but the treatment with IESLs restored them in the OVX+IESL group (Figure 4b).

## 4. Discussion

In the present study, we investigated the effects of IESL in OVX mice to determine how IESL influences cognitive decline in postmenopausal women. The OVX mouse is commonly used as a postmenopausal animal model, and it has been reported that ovariectomy induces poor metabolic phenotypes [32,33]. In this study, we observed that ovariectomy significantly increased the body weight and fat accumulation, but there was no significant difference in food intake among the groups. In humans, it was reported that hormonal change caused by menopause increased body weight and abdominal obesity and contributed to physical and psychological disorders [34]. Metabolic abnormalities, such as insulin resistance and visceral obesity, induce oxidative stress, dyslipidemia, and inflammation, and all of these are risk factors for Alzheimer’s disease, which mainly exhibits cognitive and memory impairment as a symptom [2,35].

In this study, we also observed that ovariectomy and administration of IESLs changed ER expression in the hippocampal homogenates. Estrogen is well known as an essential regulator of neuronal cell survival, proliferation, and plasticity [36,37]. In addition, phytoestrogens, including isoflavones, bind to ER α and β and activate gene transcription via ER-mediated pathways [38]. In the previous study, we confirmed that IESL contains various types of isoflavones [23], such as daidzein and genistein, by high-performance liquid chromatography [39], and it was widely reported that these isoflavones play an essential role in relieving various postmenopausal symptoms [39,40]. Estrogen receptors are distributed in multiple brain subregions, including the hippocampus, and are involved in hippocampus-dependent functions [11]. In the female rat hippocampus, expression of ER β significantly decreased with aging, but the treatment of estradiol increased synaptic ER β expression in the CA 1 region [41]. In addition, the change in ER expression according to aging or menopause and the ratio of ER α and ER β that interacts with estradiol are essential for cognitive function or memory improvement [11]. In the present study, we did not check estrogen levels in the IESL-treated mice; however, it has been reported that isoflavone treatment did not alter serum estradiol levels in perimenopausal women [42]. Isoflavones have estrogenic activity and can bind to both alpha and beta estrogen receptors [43]. ER α- and ER β-mediated estrogen signalings are involved in synaptogenesis and hippocampal functions [44], and it was also reported that the administration of soy isoflavones ameliorated cognitive dysfunction in Alzheimer’s disease.

Aging and menopause can cause brain atrophy and a decline in cognitive function [45]. In animal studies, loss of estrogen and female hormone therapy has induced structural changes in brain subregions and altered hippocampal functions, including gene expression, learning and memory, and synaptic plasticity [46]. Hippocampal atrophy induced by menopause significantly impairs not only spatial learning and memory but processing speed [46]; however, estrogen therapy restored hippocampal volume, and estrogen receptor-mediated pathways are deeply associated with maintaining hippocampal function [47,48,49].

The MWM and Y-maze tests were performed to investigate whether the administration of IESLs ameliorated the memory deficits induced by ovariectomy in the present study. As expected, in the MWM test, the OVX group had increased escape latencies and swimming distances during the training trial and reduced the time spent in the target quadrant. Similarly, the OVX group had significantly decreased spontaneous alternation in the Y-maze test. However, treatment with the IESL extracts restored the indicators of the MWM test and decreased the spontaneous alternation in the Y-maze test compared to the OVX group. The hippocampus is closely involved in performing tasks about visuospatial working memory or reference memory [50,51], and treatment with IESLs significantly enhanced the spatial working memory and reference memory in both tests. These results suggest that IESLs can restore hippocampal function impaired by ovariectomy. In addition, women in transition to menopause frequently suffer from mood disorders [52]. The ER β-selective modulator decreased anxiety and depressive behavior in the OVX rats [53], and the failure of ER β impaired the expression of brain-derived neurotrophic factors critically implicated in cognition and mood [54,55]. However, in the present study, we did not observe depression-related behavior in the SPT test.

Various diseases, such as metabolic disorders, neurodegenerative diseases, or physiological changes, including aging and menopause, exhibit cognitive decline as a common symptom, and many studies have reported that the PI3K/Akt signal pathway is involved in restoring the cognitive decline induced by the diseases [56,57,58]. Previous studies reported that activation of the PI3K/Akt signaling significantly improved depression-related symptoms in the perimenopausal model [59]. Signaling mediated by the G-protein coupled estrogen receptor 1, recently targeted for estrogen-related cancer therapy, alleviated cognitive impairment induced by traumatic brain injury via the PI3K/Akt pathway [60]. Low estrogen levels cause inflammatory processes due to menopause, which is closely associated with the pathogenesis of cognitive impairment and dementia [61]. In addition, estrogens, well known as neuroprotective agents, regulate inflammasomes produced by stroke and depressive disorders in the brain and reduce neuronal death and the occurrence of psychiatric problems [62]. In the hippocampus, acute administration of estradiol prevented CA1 pyramidal neurons from apoptosis induced by ischemic damage via activating the PI3K/Akt signaling [63,64]. In the present study, we confirmed that ovariectomy significantly decreased the PI3K/Akt expression, and administration of IESLs restored it. Expression of the ER β decreased by ovariectomy was also markedly reversed by administration of IESLs. Combining these results with the behavioral experiments may suggest that phytoestrogens of the IESLs are involved in estrogen receptor-mediated signaling, and cognitive function is restored by the estrogen receptor-PI3K/Akt pathway in the hippocampus.

## 5. Conclusions

The present study showed the effects of IESLs on the cognitive function in OVX mice. In behavioral tests, ovariectomy impaired cognitive functions, but administration of IESLs restored it. In addition, in the OVX mice, treatment of IESLs restored ERs and PI3K/Akt expression. The positive effects of IESLs on cognitive functions may be closely related to ER-mediated PI3K/Akt signaling pathway in the hippocampus.

## Data Availability

Not applicable.
